# In-vitro generation of follicle-like structures from human germ cell-like cells derived from theca stem cell combined with ovarian somatic cells

**DOI:** 10.1186/s13048-023-01315-x

**Published:** 2024-01-02

**Authors:** Seyedeh Nasim Mirbahari, Christiani A. Amorim, Fatemeh Hassani, Mehdi Totonchi, Mahnaz Haddadi, Mojtaba Rezazadeh valojerdi, Azam Dalman

**Affiliations:** 1 Department of Developmental Biology, School of Basic Sciences and Advanced Technologies in Biology, University of Science and Culture, Tehran, Iran; 2 Department of Embryology, Reproductive Biomedicine Research Center, Royan Institute for Reproductive Biomedicine, ACECR, Banihashem Avenue, Resalat Highway, PO Box, Tehran, 19395- 4644 Iran; 3 Pôle de Recherche en Physiopathologie de la Reproduction, Institut de Recherche Expérimentale et Clinique, Université Catholique de Louvain, Brussels, Belgium; 4 Department of Genetics, Reproductive Biomedicine Research Center, Royan Institute for Reproductive Biomedicine, ACECR, Tehran, Iran; 5 Department of Anatomy, Faculty of Medical Sciences, Tarbiat Modares University, Tehran, Iran

**Keywords:** Human theca stem cells, Germ cell-like cells, Primordial follicle-like structure, In vitro development

## Abstract

**Background:**

The objective of this study was to induce the differentiation of human theca stem cells (hTSCs) into germ cell-like cells (hGCLCs) and assess their developmental progression following in vitro 3D culture with ovarian somatic cells within the follicle-like structures. To achieve this, the hTSCs were isolated from small antral follicles of three patients of varying ages and were then seeded in a differentiation medium for 40 days. The differentiated hGCLCs were subsequently aggregated with somatic ovarian cells (cumulus cells and hTSCs) in a ratio of 1:10 and cultured in a growth medium in a suspension culture dish. In addition to examining the morphologies, sizes, and viabilities of the differentiated hGCLCs, this study also analyzed the expression of DAZL and GDF9 proteins within the follicle-like structures.

**Results:**

After 12 days, the hTSCs began to differentiate into hGCLCs, with their shapes changing from spindle-shaped to spherical. The sizes of hGCLCs increased during the differentiation period (from 25 μm to 50 μm). The survival rate of the hGCLCs after differentiation and in vitro development in primordial follicle-like structures was 54%. Unlike hTSCs, which did not express the DAZL protein, the hGCLCs and follicle-like structures successfully expressed DAZL protein (*P*-value < 0.05). However, hGCLCs poorly expressed the GDF9 protein. Further, the culture of hGCLCs in primordial follicle-like structures significantly increased GDF9 expression (*P*-value < 0.05).

**Conclusion:**

In conclusion, our study demonstrated that 3D cultures with ovarian somatic cells in follicle-like structures caused the successful differentiation of reproducible hGCLCs from hTSCs derived from three patients of different ages. Moreover, this method not only enhanced the in vitro development of hGCLCs but also presented a novel approach for co-culturing and developing in vitro oocyte like cells, ultimately leading to the production of artificial follicles.

## Background

Cytotoxic methods, such as chemotherapy and radiotherapy, are common cancer treatments [1]. Although these therapies increase patient survival rates, they may irreparably damage ovarian follicles, leading to premature ovarian insufficiency and potentially increasing abortion rates, health risks, and psychological conditions associated with early menopause [2]. In this context, oncofertility seeks to develop appropriate methods to maintain or improve fertility in cancer patients and those with reproductive function threatened by disease.

Among the numerous strategies under development, the in vitro generation of new oocytes has gained momentum in recent years. Studies have shown that oocyte-producing stem cells isolated from women's ovaries can differentiate into oocytes [3]. Oocytes differentiated from ovarian stem cells retrieved from mice were suitable for fertilization and implantation, as evidenced by embryo development and live births [4]. Thus, therapeutic manipulation of adult stem cells could potentially overcome infertility and prevent ovarian failure.

One source of somatic stem cells in the ovarian cortex is the theca layer, with theca stem cells (TSCs) first recognized in 2007 in mice. These cells demonstrated signs of differentiation into early precursor steroidogenic cells in vitro [3]. The three different types of theca cells, which are responsible for generating distinct subtypes, are derived from specific lineages known as structural, androgenic, and perifollicular cells. Additionally, their potential lineage-negative precursor has also been considered in understanding their cellular origins and differentiation hierarchy [5]. TSCs have been described in the ovaries of other animal species, such as pigs and sheep [6, 7]. Adib et al. [6] characterized TSCs containing mesenchymal stem cells and pluripotent stem cells in sheep and showed that sheep TSCs could differentiate into osteocyte, adipocyte, theca progenitor-, and oocyte-like cells. Porcine TSCs have been shown to differentiate into the mesenchymal lineage and oocyte-like cells, as indicated by their expression of oocyte-specific genes (GDF9B, C-MOS, DAZL, VASA, ZPC, SCP3, and STEIIA) after differentiation into oocytes [7].

Human theca stem cells (hTSCs) have also been characterized in small antral follicles and have been shown to have mesenchymal characteristics, such as surface markers CD29, CD44, CD73, CD90, and CD105, and differentiation into adipocyte-, osteocyte-, and chondrocyte-like cells [8]. These cells successfully differentiated into oocyte-like cells in our previous study [9]. However, there is limited information available regarding the developmental potential of these differentiated cells. Therefore, it is important to investigate the production of functional oocytes from both germ and somatic stem cells, as well as to advance ovarian follicle culture techniques. Thus, this study aims to evaluate the developmental process of hGCLCs after their in vitro 3D culture with ovarian somatic cells.

## Results

### Evaluation of morphology and viability of hTSCs

**Fig. 1:**
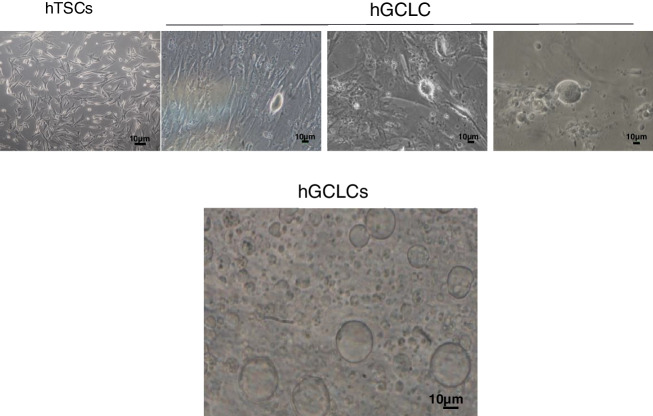
Phase-contrast images of hTSCs and differentiated hGCLCs in in vitro culture

The hTSCs in the culture were spindle-shaped fibroblast-like after a week, and they could adhere to the bottom of the culture dish (Fig. 1). Staining of these cells before differentiation using Trypan Blue showed that after 40 days of cell culture, on average, 84.96% of the cells were alive.

### Evaluation of morphology, size and viability of hGCLCs

**Fig. 2:**
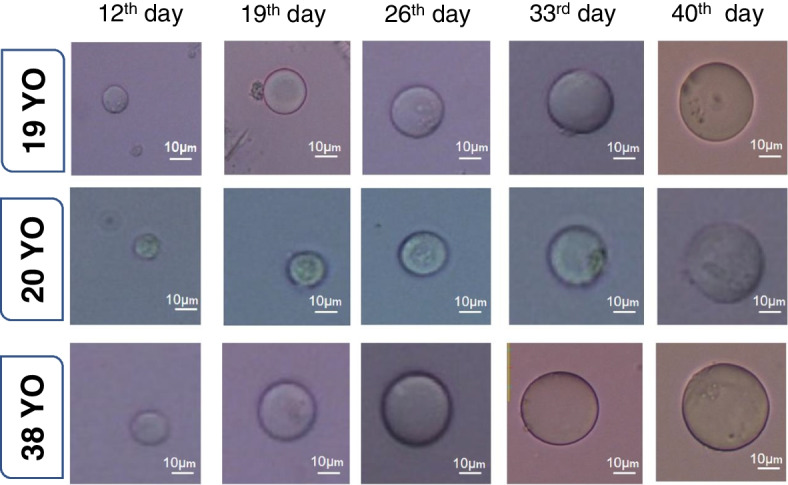
HGCLC size and morphology on different days of in vitro culture. These cells reached approximately 50 µM on day 40 in the differentiation medium

hTSCs acquired from the three patients were cultured in DMEM/F12 supplemented with 5% FBS, 5% human follicular fluid, 0.23 mM sodium pyruvate (Sigma, Japan), 0.1 mM non-essential amino acids (Sigma, USA), 2 mM L-glutamine, and 0.1 mM β-mercaptoethanol (Sigma, USA) for 40 days. This medium was changed twice a week. Around the 12th day of differentiation, the shapes of a few cells changed from spindle to spherical (approximately 2%). The morphologies of the spherical cells were similar to that of an oocyte. Following differentiation, the size and number of hGCLCs increased significantly over time (from an initial diameter of 20 to 25 μm to 50 μm, Fig. 2). The mean survival of hGCLCs was 73.32% on the 40^th^ day.

### Evaluation of DAZL and GDF9 protein expression in hGCLCs

**Fig. 3:**
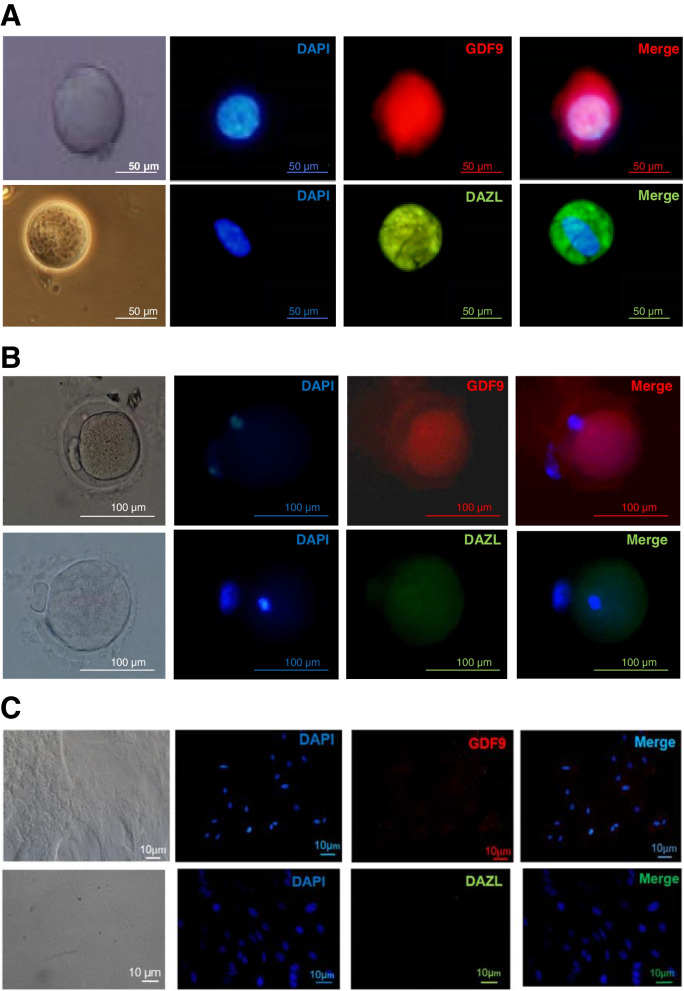
Immunofluorescence staining of DAZL and GDF9 for negative and positive controls. Mouse GV (**A**) and MII oocytes (**B**) as a positive control and hTSCs as a negative control (**C**). a. Phase-contrast image of mouse oocytes and hTSCs. b. Mouse oocytes and hTSCs after staining the nuclei with DAPI. c. Immunofluorescence staining of GDF9 and DAZL. d. Merged DAPI and primary antibody-secondary antibody-FITC staining of GDF9 and DAZL. Scale bars (**A**): 50 μm, (**B**): 100 μm and (**C**): 10 μm

**Fig. 4:**
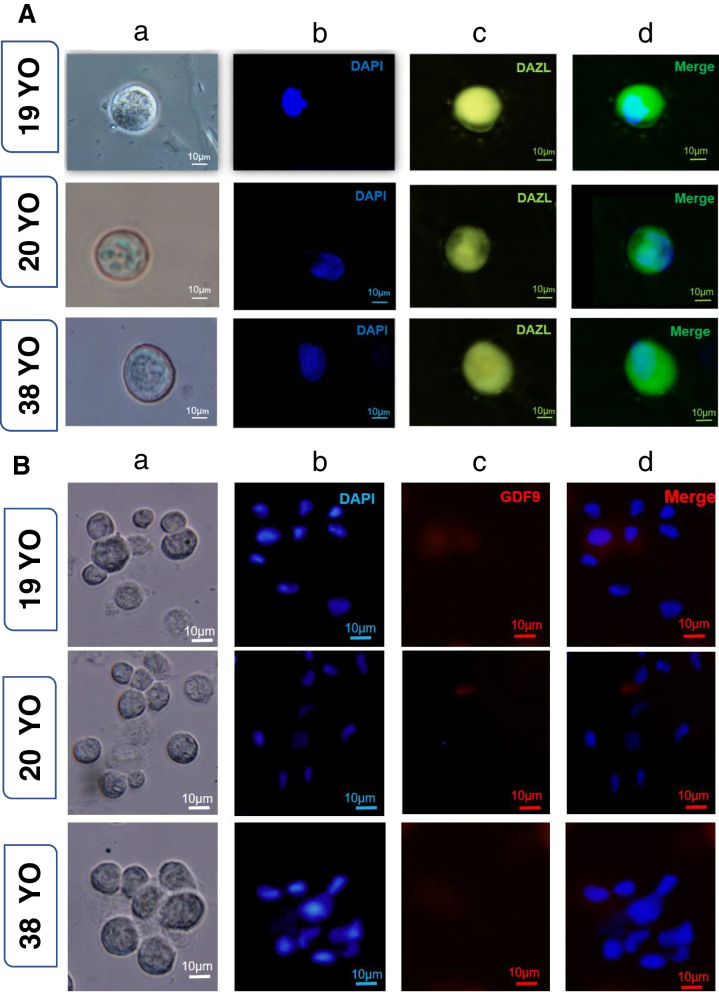
HGCLC staining. **a** Phase-contrast image of hGCLCs. **b** hGCLCs after staining with DAPI. **c** Immunofluorescence staining of DAZL (**A**) and GDF9 (**B**) of the same cells. **d** Merged DAPI and primary antibody-secondary antibody-FITC staining of DAZL and GDF9. Scale bars: 10 μm

To evaluate the in vitro development of hGCLCs, the expression of DAZL and GDF9 proteins in these cells was assessed. hTSCs and mouse oocytes (GV and MII) were used as negative and positive controls, respectively (Fig. 3). Immunocytochemical evaluation of DAZL protein showed that this protein was highly expressed in each hGCLCs in all three patients (Fig. 4A). However, GDF9 protein expression was very low in all hGCLCs that were obtained from three patients (Fig. 4B).

### Evaluation of morphology and size of follicle-like structures

**Fig. 5:**
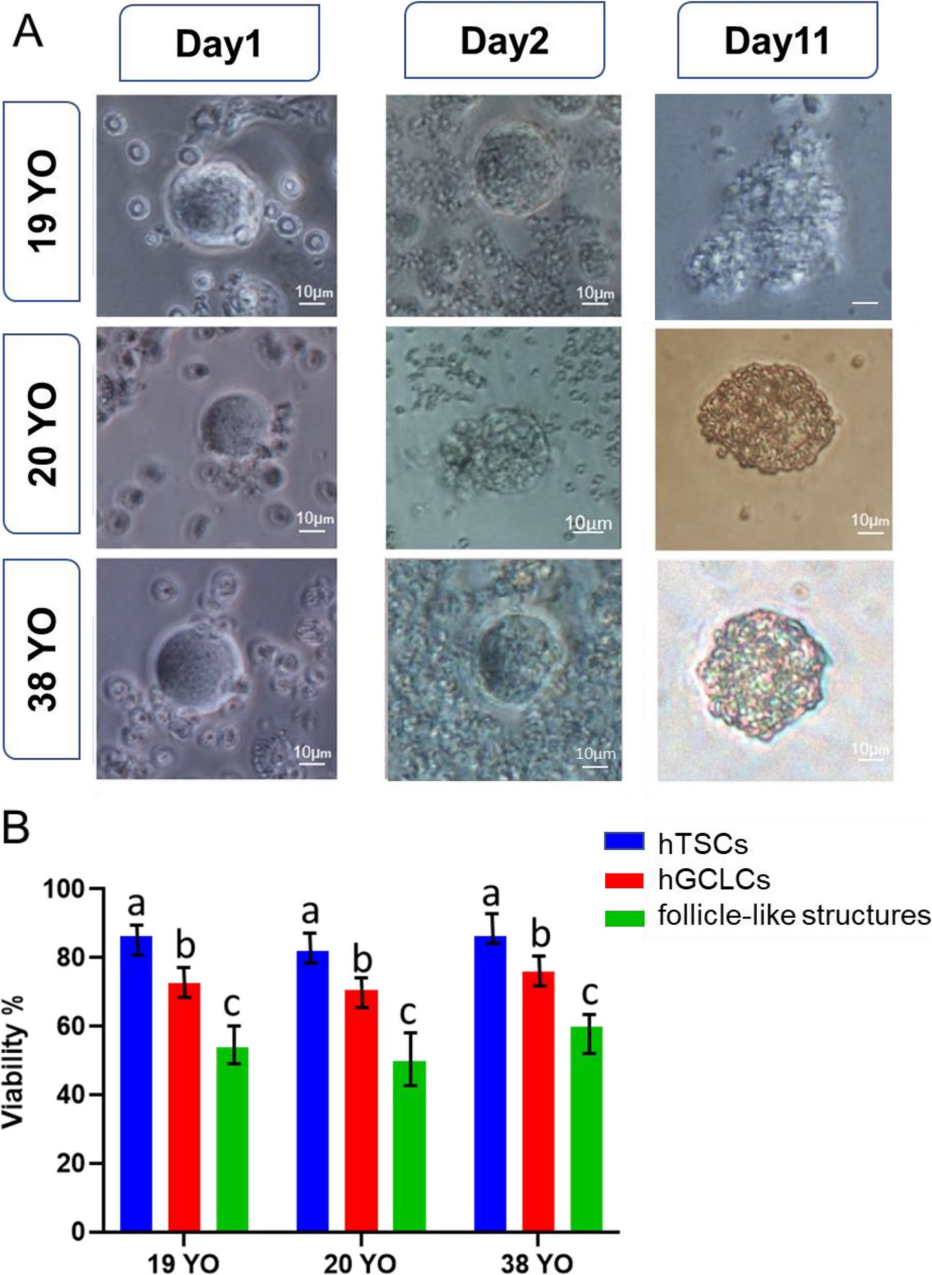
Cell aggregates. **A** Arrangement of somatic cells around in vitro-grown hGCLCs from all three patients cultured on days one, two, and 11. **B** Viability of hTSCs, hGCLCs and follicle-like structures. Values with different superscripts are significantly different. (*P*-value <0.05)

After differentiation and growth, the hGCLCs were placed along with somatic cells (hTSCs + cumulus cells) in suspension culture dishes to grow under the influence of factors secreted by these cells. Since somatic cells attach to the bottom of the dish for survival, we suspect that somatic cells use the floating hGCLCs as a substrate for attachment. Therefore, after 24 h, the somatic cells moved toward the hGCLCs and supported these cells. The number of somatic cells around hGCLCs increased during culture. On the 11th day, follicle-like structures were observed, and somatic cells were wholly attached to the hGCLCs (Fig. 5A). The number of somatic cells around each follicle significantly increased with every passing day. After 11 days of culture, the size of hGCLCs could not be measured due to the binding of somatic cells around GLC. At the end of this period, the average cell survival was 54.66% (Fig. 5B).

### Evaluation of GDF9 and DAZL protein expression in follicle-like structures

**Fig. 6:**
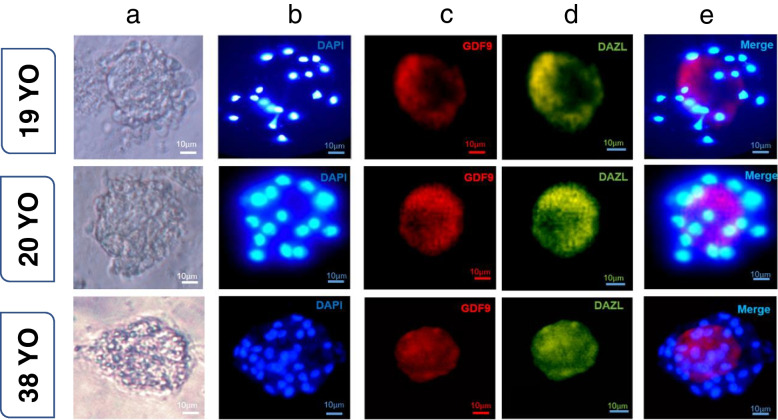
Immunofluorescence staining of GDF9 and DAZL in follicle-like structures of three patients. **a** Phasecontrast image of follicle-like structures. **b** Follicle-like structures after the nuclei are stained with DAPI. **c** Immunofluorescence staining of GDF9. **d** Immunofluorescence staining of DAZL. **e** Merged images of DAZL and GDF9 antibodies with DAPI. Scale bars: 10 μm

**Fig. 7:**
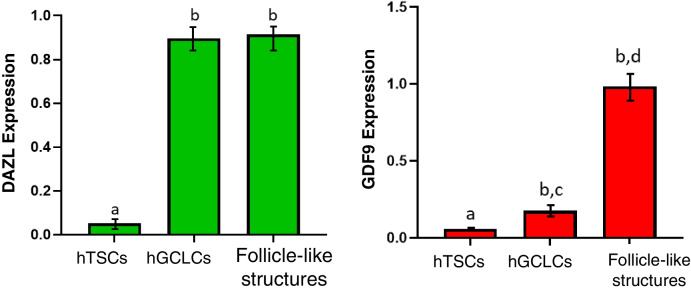
Quantitative expression of DAZL and GDF9 proteins in the hGCLCs and follicle-like structuresgroups. Values with different superscripts (**a** with **b**) and (**c** with **d**) are significantly different (*P*-value < 0.05)

At the end of the culture period, all the follicle-like structures that were subjected to the staining process expressed the GDF9 protein well. Additionally, it was shown that these cells were capable of expressing the DAZL protein, similar to hGCLCs (Fig. 6). Quantitative analysis of the data revealed a significant increase in the expression of DAZL and GDF9 proteins in hGCLCs and follicle-like structures compared to the control group (hTSCs) (Fig. 7, *P*-value < 0.05). While no significant difference was observed in the expression of DAZL between the groups of hGCLCs and follicle-like structures, a significant difference in the GDF9 protein was observed (*P*-value < 0.05).

## Discussion

In our previous study, we focused on the isolation and differentiation of hTSCs into hGCLCs [9]. These cells expressed germ cell genes like *PRDM1, PRDM14, VASA, DAZL*, and *OCT4*, oocyte-specific genes such as *ZP1, 2, 3* and *GDF9*, and meiotic-specific markers like *SCP3* and *DMC1*. In addition, GDF9, OCT4, DAZL, VASA, and ZP3 proteins were present in hOLCs [9]. However, it was necessary to study more patients to ensure the reproducibility of the results. In this study, we were able to successfully differentiate hGCLCs from hTSCs in three patients across various age groups in the presence of human follicular fluid. Various concentrations of hormones, including FSH, LH, estradiol and progestins, proteins, reactive oxygen species, cytokines as well as growth factors are present in the human follicular fluid. Recognizing the inherent variability among individuals and with the goal of enhancing the reproducibility of our approach, we have pooled follicular fluid samples from various patients. The differentiated hGCLCs were able to form three-dimensional structures similar to those of follicles in a laboratory setting using ovarian cells. These follicle-like structures showed good expression of DAZL and GDF9 proteins. Previous studies have suggested that proliferative interstitial cells present in the ovarian stroma possess stem cell characteristics [10]. These somatic stem cells can differentiate into primordial germ-like cells and eventually into oocyte-like cells expressing the DAZL marker [11]. Our previous studies also indicated that TSCs found in the ovaries of sheep and humans possess stem cell characteristics [6, 8]. Given the morphological similarity and gene and protein expression patterns, we suggest that hTSCs could differentiate into oocyte-like cells [9]. Interestingly, the expression of germ cells and certain oocyte-specific genes indicates that these cells appear to be in the process of transitioning from germ cell to the primary oocyte. To further explore the developmental potential of these cells, we used reaggregation techniques to create follicle-like structures with ovarian cells. This approach has been previously reported to be effective in replacing defective oocytes or somatic cells [12]. In a study by Eppig et al., somatic cells derived from the ovaries of LTXBO mice induced the growth of an isolated oocyte from the ovaries of B8SJL mice in a reaggregated ovary structure. After transplantation and 19 days of growth, the somatic cells around the oocyte created an antral follicle-like structure [13].

In the present study, somatic cells attached into hGCLCs one day after being placed in suspension culture. A previous study reported attachment of somatic cells to germ cells after two days [14]. Structures similar to those in previous studies [12, 13, 15], were observed at the end of the seventh day, with somatic cells completely attached to hGCLCs. More than 80% of hGCLCs were retained in reaggregated follicles for 11 days in vitro. However, after prolonged in vitro culture, survival rates decreased to 54.66% due to cellular stress caused by culture conditions or metabolite accumulation [16]. Ovarian cells were added to the culture at a rate of 10 cells per hGCLC on the first day and progressively increased over time resulting in the formation of at least two cell layers around hGCLCs by the eleventh day. Cell proliferation was used as a criterion for cell survival and to determine the oocyte's developmental stage in the reaggregated ovary in previous studies [13, 15].

At the end of the 11th day of co-culture, DAZL protein expression follicle-like structures showed a tendency to increase compared to before exposure to ovarian cells, although this difference was not significant.

GDF9 protein expression was evaluated to determine cell development in the structure of the follicle-like structures and to check if this differed from differentiated hGCLCs. GDF9 protein levels significantly increased in the follicle-like structures compared to hGCLCs. The GDF9 protein can be detected in oocytes of primordial follicles onwards in the human ovary and plays an important role in folliculogenesis [11, 17]. Hubner et al. differentiated mouse embryonic stem cells into oocytes and confirmed this process using the GDF9 oocyte marker [18]. Moreover, the GDF9 gene and protein expressions were used to ensure the differentiation of sheep theca stem cells (TSCs) into ovarian-like cells (OLCs) [6]. In our previous study, we demonstrated by real-time PCR that the co-cultivation of theca stem cell-derived cells with granulosa cells leads to an upregulation of GDF9 expression [9]. In this study, we used three-dimensional culture conditions alongside hTSCs and cumulus cells to create an in vitro follicle-like structures to mimic the in vivo environment. Cumulus cells secrete factors which promote follicular growth, oocyte maturation, sterol biosynthesis, and regulate oocyte gene transcription [19]. Furthermore, such cells provide the required energy to initiate oocyte meiosis and boost glycolysis [20]. While the cumulus cells used in our study could be already in the process of luteinization, our findings demonstrate that they still secrete factors that play an important role in oogenesis. Indeed, Shah et al. [21] successfully differentiated embryonic stem cells into germ lineage using a conditioned medium from cumulus cells aspirated from cumulus-oocyte complexes after oocyte in vitro maturation. The differentiated oocyte-like cells expressed ZP4 protein and progressed embryo-like structures in expanded culture [21].

It appeared that hGCLCs had thin ZP in follicle-like structures. It is possible that the zona pellucida in these cells has not yet formed properly, or their fibers have not separated from the membranes of the hGCLCs. This requires further evaluation.

In our current study, we isolated hTSCs from the ovaries of women of different ages and achieved similar and promising results in all cases.

## Conclusion

In general, morphological similarities, size, and protein expression showed that reproducible hGCLCs could be differentiated from hTSCs in three patients of different ages. The association of somatic ovarian cells (cumulus and hTSCs) with hGCLCs in three-dimensional conditions caused further development, so that the follicle-like structures could increasingly express the GDF9 protein, which is expressed from the primordial follicle stage in the oocyte. However, the trans-zonal projections of cumulus cells and hGCLCs are not yet clear. Further investigation, including histological and immunostaining evaluation for some proteins (i.e., connexins and transzonal projections), is needed to demonstrate an association between cumulus cells and hGCLCs. It is also possible that the interaction between cumulus cells and hGCLCs may lead to the differentiation of hTSCs into theca cells since GDF9 expression in oocytes organizes a cascade of events leading to its recruitment and transformation. However, this requires further clarification.

The overall sizes of these cells and the surrounding compartments are comparable to those of the primordial follicle. This model paves a way for further laboratory studies on the exact mechanism of germ cell formation and development. We are currently conducting another ongoing study to analyze the transcriptome profile of differentiated hGCLCs. However, their functionality requires further testing.

## Methods

### Human Theca Stem Cell Isolation and Culture

**Fig. 8:**
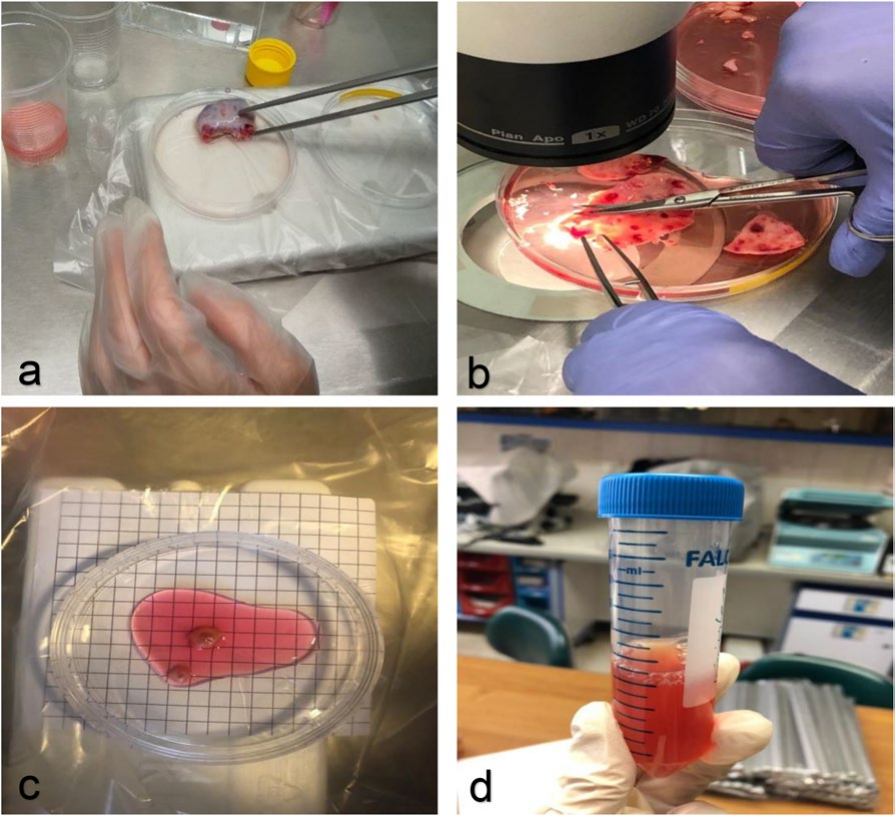
Stages of isolation of human small antral follicles from ovaries. **a** Ovary isolated from patients. **b** Cutting and scratching the ovary. **c** Follicle size 3-5 mm. **d** Enzymatic digestion of follicles

In addition to the hTSCs obtained from the ovary of a 19-year-old patient that was evaluated in the previous study, ovaries from two transgender patients aged 20 and 38 years were collected to ensure reproducibility of the test at different ages. Small antral follicles (3–5 mm) were collected from the three ovaries. Three different age ranges were used in this experiment to evaluate the reproducibility of differentiation of hTSCs into hGCLCs at young and late reproductive ages. The follicles were cut in half, and the oocytes and granulosa cells were removed using a spatula, leaving only the theca layer in the follicle wall. The isolated layer was dissected into small pieces and incubated with 0.5% collagenase type I (Sigma, St. Louis, USA) at 37°C. After 45 min, DMEM/F12 ( Gibco, Grand Island, NY, USA), containing 10% fetal bovine serum albumin (FBS, Gibco, USA) was added to neutralize the enzyme activity. The cell suspension was further purified by passing it through 100 and 40 µm filters (BD Falcon, Mexico) [7], and then cultured in DMEM/F12 supplemented with 10% FBS, 2 mM glutamine (Gibco, UK), 100 U/mL penicillin G (Sigma, USA), 100 µg/mL streptomycin (Sigma, USA), 20 ng/mL epidermal growth factor (EGF; Royan Biotech, Tehran, Iran), 10 ng/mL basic fibroblast growth factor (bFGF; Royan Biotech, Tehran, Iran), and 10 ng/mL glial cell line-derived neurotrophic factor (GDNF; Royan Biotech, Tehran, Iran) at 37°C in a 5% CO2 incubator. Cells that reached 70% confluence in the third passage were considered hTSCs (Fig. 8).

### Preparation of human follicular fluid

Follicular fluid samples were obtained from four ICSI-treated patients (age range: 20–38 years) with male factor infertility and were centrifuged at 2000 rpm for 10 min to remove cells. The cell supernatants were subjected to incubation at 56°C for 30 min to inactivate enzyme function, followed by storage at -20°C.

### Differentiation of hTSCs to hGCLCs

To differentiate hTSCs to hGCLCs, 7 × 10^4^ cells were seeded in DMEM/F12 supplemented with 5% FBS, 5% human follicular fluid, 2 mM L-glutamine, and 2 mM penicillin–streptomycin (Sigma) for 40 days [22]. The medium was refreshed every three days, and cells that became spherical were isolated using a Pasteur pipette and considered hGCLCs, as previously established in our study [9].

### Preparation of cumulus cells

Cumulus cells were obtained from three patients who underwent ICSI treatment at the Royan Institute. The age range of these patients was 19 to 38 years, and they were diagnosed with male factor infertility. Following oocyte retrieval, the cumulus masses were carefully separated from COCs using hypodermic needles and treated with 300 μg/ml hyaluronidase for 30 s. Dissection medium (Minimum essential medium alpha (α-MEM, Sigma, St Louis, MO, USA)) supplemented with 10% FBS was added for enzyme inactivation, and cells were further washed using centrifugation at 200 × g for 10 min. The pellets were cultured for one day in DMEM/ F12 supplemented with 10% FBS, 2 mM glutamine (Gibco), 100 U/mL penicillin G (Sigma), and 100 µg/mL streptomycin (Sigma), and incubated at 37°C and 5% CO2 [23].

### Follicle-like structures

This experiment aimed to determine whether placing hGCLCs next to somatic cells would increase the developmental potential of hGCLCs. To accomplish this, hGCLCs and somatic cells (hTSCs and cumulus cells) were combined. One-tenth of germ cells and all somatic cells, with equal proportions of cumulus cells and hTSCs, were used to make follicle-like structures. Phytohemagglutinin (PHA-P; Sigma) was added to create a final concentration of 35µg/mL to promote cell cohesion. The culture media was Waymouth MB752/1 (Gibco), supplemented with 0.23 mM pyruvic acid (Sigma, Japon), 50 mg/L streptomycin sulfate, 75 mg/L penicillin-G (Sigma), and 10% FBS. Samples were cultured at 37°C in an atmosphere of 5% CO2 and 95% air. Each 10 µl droplet of a 6-cm suspension culture dish contained five follicle-like structures [10].

### Evaluation of the viability of hTSCs, hGCLCs and follicle-like structures

Trypan blue staining was used to assess cell viability. Living cells are impermeable to trypan blue, whereas dead cells absorb it. A 0.4% concentration of trypan blue was mixed with an equal volume of the cell suspension and added to a well in a 96-well plate. The mixture was gently pipetted to ensure proper distribution. For hTSCs, 10 μl of the stained cell suspension was placed on a hemocytometer slide to count the cells.

### Evaluation of size and morphology of hTSCs, hGCLCs and follicle-like structures

The transition in morphology from a spindle shape to a spherical shape, as well as the size of hGCLCs and follicle-like structures, were assessed at various stages of differentiation. These evaluations were conducted using an inverted microscope, and measurements were recorded.

### Evaluation of DAZL and GDF9 protein expression using immunocytochemistry

hGCLCs and follicle-like structures were evaluated for the DAZL and GDF9 proteins, specific markers for germ cells and oocyte. To confirm that the antibodies were specific for DAZL and GDF9 proteins, the secondary antibodies were used alone as negative controls. Images were taken using a fluorescence microscope (Eclipse 50i; Nikon, Japan) and analyses and merge were performed with ImageJ Tool- kit software (version 1.46r, National Institutes of Health) and Java software executing on 32-bit architecture (version 1.6.0–20, Oracle). hTSCs and mouse oocytes (GV and MII) were used as negative and positive controls, respectively.

Samoles were fixed with a 4% paraformaldehyde solution for 30 min and permeabilized with 0.05% Triton X-100 in PBS for 10 min at room temperature. The cells were then incubated overnight at 4°C with DAZL (rabbit polyclonal, Abcam, city, USA, 1:200 (in blocking buffer) and GDF9 (goat polyclonal antibody, Santa Kruz, USA, 1: 100 ratio). The samples were washed with a PBS/Tween solution (0.05%) for 15 min. The cells were incubated for one hour at 37°C with FITC rabbit anti-goat secondary antibody at a 1:200 dilution for DAZL antibody and FITC goat anti-donkey secondary antibody at a 1:200 dilution for GDF9 antibody. Nuclei were stained with 1 µg/mL 4’,6-diamidino-2-phenylindole (DAPI, Sigma, USA) for 5 min. For negative controls of staining, the secondary antibody was used alone. The image intensity was quantified using ImageJ software (version 1.42q, Wayne Rasband, NIH, Bethesda, USA).

### Statistical analysis

Statistical analysis was performed using the SPSS software version 22.0. Continuous variables were expressed as a mean ± standard deviation, while stratified variables were expressed as numbers (percentage). The Kolmogorov–Smirnov test was used to check the normality of the studied variables. Due to the groups' dependence, a repeated measures test was used to examine the three groups and the corresponding post hoc tests were used for significant variables. Paired t-tests were used to evaluate the two groups. A p-value of less than 0.05 was considered statistically significant.

## Data Availability

Data sharing is not applicable to this article as no datasets were generated or analysed during the current study.

## Abbreviations

3D: Three-Dimensional
α-MEM: Minimum Essential Medium Alpha
bFGF: Basic Fibroblast Growth Factor
CD: Cluster of Differentiation
C-MOS: C-Yes Oncogene Protein
COCs: Cumulus-Oocyte Complexes
DAPI: 4’,6-Diamidino-2-Phenylindole
DAZL: Deleted in Azoospermia-Like
DMEM/F12: Dulbecco's Modified Eagle Medium/Nutrient Mixture F-12
EGF: Epidermal Growth Factor
FBS: Fetal Bovine Serum
FITC: Fluorescein Isothiocyanate
GDF9: Growth Differentiation Factor 9
GDF9B: Growth Differentiation Factor 9B
GDNF: Glial Cell Line-Derived Neurotrophic Factor
GLCs: Germ-Like Cells
GV: Germinal Vesicle
hGCLCs: Human Germ cell-Like Cells
hTSCs: Human Theca Stem Cells
ICSI: Intracytoplasmic Sperm Injection
MII: Metaphase II
OCT4: Octamer-Binding Transcription Factor 4
PBS: Phosphate-Buffered Saline
PHA-P: Phytohemagglutinin
PRDM1: PR Domain Zinc Finger Protein 1
PRDM14: PR Domain Zinc Finger Protein 14
SCP3: Synaptonemal Complex Protein 3
SPSS: Statistical Package for the Social Sciences
STEIIA: Spermatogenesis and Oogenesis Specific Basic Helix-Loop-Helix Transcription Factor 2
VASA: DEAD-Box Helicase 4
ZPC: Zona Pellucida C
